# The role of native language and beat perception ability in the perception of speech rhythm: Native language, beat perception, and speech rhythm perception

**DOI:** 10.3758/s13423-024-02513-4

**Published:** 2024-07-19

**Authors:** Eline A. Smit, Tamara V. Rathcke

**Affiliations:** Department of Linguistics, University of Konstanz, Konstanz, Germany

## Abstract

The perception of rhythm has been studied across a range of auditory signals, with speech presenting one of the particularly challenging cases to capture and explain. Here, we asked if rhythm perception in speech is guided by perceptual biases arising from native language structures, if it is shaped by the cognitive ability to perceive a regular beat, or a combination of both. Listeners of two prosodically distinct languages - English and French - heard sentences (spoken in their native and the foreign language, respectively) and compared the rhythm of each sentence to its drummed version (presented at inter-syllabic, inter-vocalic, or isochronous intervals). While English listeners tended to map sentence rhythm onto inter-vocalic and inter-syllabic intervals in this task, French listeners showed a perceptual preference for inter-vocalic intervals only. The native language tendency was equally apparent in the listeners’ foreign language and was enhanced by individual beat perception ability. These findings suggest that rhythm perception in speech is shaped primarily by listeners’ native language experience with a lesser influence of innate cognitive traits.

## Introduction

Rhythm is considered an essential aspect of temporal auditory signals such as music and speech, potentially being universal across languages and cultures (Patel & Iversen, 2014; Nettl, 2000; Kotz et al., 2018; Savage et al., 2015). In linguistic research, rhythm is, however, a hotly – and controversially – debated topic (Nolan & Jeon, 2014). In music, rhythm can be described as structured time intervals between discrete acoustic events representative of the overall temporal structure (Cameron et al., 2015). It is sometimes defined as referring “to the temporal patterns created by the onsets and durations of acoustic events in an incoming sequence” (Fiveash et al., 2022, p.4). Under this view, rhythm is certainly applicable to speech which, similar to music, has a highly structured temporal dimension (Turk & Shattuck-Hufnagel, 2014). Such temporal structure is known to be crucial to the perception of auditory signals and their subsequent higher-level processing in the brain (Fiveash et al., 2021; Kraus & Chandrasekaran, 2010; Tierney & Kraus, 2014).

In music, rhythm is typically represented as a hierarchy comprised of layers of nested temporal structures, with the lowest level being the level of a beat (Lehrdahl & Jackendoff, 1983). The cognitive ability to perceive a regular beat (or a pulse) in auditory signal is referred to as beat perception (Merchant et al., 2015; Honing, 2013; Large & Palmer, 2002). This ability is important for a number of musical and social behaviors, including dancing and joint music-making. The majority of people have been found to be able to extract a beat from an auditory rhythm (Fiveash et al., 2022; Repp, 2010; Sowiński & Dalla Bella, 2013) and to synchronize their movements with the external beat (Merchant et al., 2015). Beat perception has thus been suggested to constitute a fundamental and possibly innate musical trait that is foundational to general rhythm perception ability (Merchant et al., 2015; Honing, 2013; Nettl, 2000), though like any cognitive skill, beat perception ability can show substantial variation among individuals (Bégel et al., 2017; Phillips-Silver et al., 2011; Bekius et al., 2016) and can be influenced by enculturation (Harrison & Müllensiefen, 2018b; Hannon & Trehub, 2005; van der Weij et al., 2017). Little is known to what extent experience with the rhythm of one’s native language may also influence rhythm perception, or if rhythm perception in speech is primarily shaped by innate cognitive processes such as beat perception ability (cf. Cameron et al., 2015) or by an interaction between both.

It has been suggested that innate perceptual processes are important for rhythm perception in auditory signals (Povel & Essens, 1985). While the timing of rhythmically relevant events can be hugely variable, it is generally assumed that human listeners can perceive only a limited number of rhythmic categories that approximate small-integer ratios of time units, in particular 1:1 and 2:1 (e.g., Jacoby et al., 2021; Jacoby and McDermott 2017). At the same time, evidence has recently been accumulating to document a pivotal role of cultural and linguistic experience in rhythm perception (Cameron et al., 2015; Zhang et al., 2020). For example, American infants prefer rhythms with a regular metrical structure over irregular rhythms while Turkish infants do not show any bias for regular rhythms, possibly because their musical culture contains both regular and irregular metrical structures (in contrast to typical Western music with a strong tendency toward metrical regularity) (Soley & Hannon, 2010). Conversely, the native language of listeners can have an influence on their perception of musical rhythm. Previous research has shown that native speakers of Japanese and English tend to perceive groupings of rhythmic tone sequences differently whereby English speakers perceived them as patterns of short–long, in contrast to Japanese speakers who perceived them as long–short. Besides, English speakers were also more variable in perception these tone sequences (Iversen et al., 2008). Japanese listeners outperform native listeners of Mandarin Chinese on musical rhythm tasks (but are outperformed by the Mandarin listeners on melody-related tasks) (Zhang et al., 2020). Moreover, experience with rhythmically distinct first and second language can enhance the perception of musical rhythms (Roncaglia-Denissen et al., 2013).

Thus, previous experience with music or language rhythms seems to shape listeners’ rhythm perception ability, though current evidence is limited primarily to rhythm perception in musical auditory sequences. Comparable evidence for the perception of spoken linguistic prompts is currently lacking and forms the motivation of the present study. A related line of research into the nature of perceptual centers (or P-centers) shows that in many languages, the perceived location of syllable beats deviates from the acoustic syllable onsets (Morton et al., 1976; Hoequist, 1983). In some languages, the P-center tends to align with vowel onsets (Marcus, 1981; Franich, 2018) while in others, the perceived beat and the acoustic syllable onset coincide (Chow et al., 2015). Previous research on the P-center location focuses primarily on mono- or bisyllabic words spoken in isolation, with little evidence as to how beat and rhythm perception arises among the complexities of natural connected speech.

Previous research tradition has assumed that the perception of speech rhythm across prosodically diverse languages is shaped by their rhythm class affiliation (Abercrombie, 1967; Pike, 1945; Ladefoged & Johnson, 1975). It divided languages mainly into two rhythm classes, “syllable-timed” with a syllable-based machine-gun rhythm (e.g., Spanish, Italian, and French) and “stress-timed” with a stress-based morse-code rhythm (e.g., English, Dutch, and Russian). Empirical studies have failed to support these assumptions (Arvaniti & Rodriquez, 2013; Arvaniti, 2009; Aubanel & Schwartz, 2020; Rathcke & Smith, 2015). An innate, or bottom-up domain of beat perception across languages, possibly due to shared neural underpinnings of rhythm in spoken language has also been suggested (Meyer, 2018; Gross & Poeppel, 2019). However, a recent cross-linguistic study examined cortical tracking of speech envelopes in English and French listeners and identified an enhanced tracking activity at the syllable level in French as compared to English listeners, suggesting the existence of a language-specific bias in beat and rhythm perception (Varghese et al., 2022).

The present study aims to shed new light on the influence that listeners’ native language and their individual beat perception ability may have on their rhythm perception in speech. Following previous research (Varghese et al., 2022; Rathcke & Lin, 2023; Rathcke et al., 2021; Merchant et al., 2015), the study tests two hypotheses:

(1) *The linguistic-bias hypothesis* predicts that English and French listeners will differ in the perceived domain of the beat and that they will transfer their language-specific perceptual bias to rhythm perception in a foreign language. Specifically, we expected French listeners to show a preference for a beat structure mapped onto syllable onsets (Varghese et al., 2022; Abercrombie, 1967; Pike, 1945), due to a high importance of the syllable for speech rhythm in this language (Goyet et al., 2013; Varghese et al., 2022). In contrast, we expected English listeners to prefer the beat to map onto vowel onsets (Rathcke & Lin, 2023; Rathcke et al., 2021), possibly because acoustic salience in English is related to changes in the acoustic qualities of vowels (Chrabaszcz et al., 2014; Zhang & Francis, 2010).

(2) *The innate cognitive skill hypothesis* predicts that listeners’ ability to perceive speech rhythm will be primarily shaped by their individual beat perception ability, regardless of their native language (Merchant et al., 2015; Honing, 2013; Nettl, 2000). Specifically, listeners with a high beat perception ability will consistently map the beat structure of speech to the acoustically shaped P-center that coincides with vowel onsets (Marcus, 1981; Franich, 2018; Rathcke & Lin, 2023) (instead of top-down imposed linguistic structures, such as syllable onsets) whereas listeners with a low beat perception ability can be expected to be highly variable in their perceptual evaluations of speech rhythm regardless of the language of stimuli (cf. Spiech et al., 2023).

## Method

### Participants

Participants were recruited via Prolific Academic platform (www.prolific.co) (Peer et al., 2017). In total, 90 native British English speakers and 118 native French speakers volunteered to take part, though only 76 British (50 female; mean age 27.84 years, range, 19–35) and 104 French (38 female; mean age 33.48 years, range, 20–67) speakers completed all tasks. Hence, the analyses reported below are based on the data of 180 participants in total. Prior to the experiment, participants filled in a background questionnaire that included questions about their linguistic and musical background. Twenty-five English and 42 French participants reported to have received musical training (ranging from minimally 2 months to maximally 27 years), though none of them were professional musicians.

As far as foreign or second language knowledge is concerned, 35 English participants reported to have studied a foreign language at school (seven of those indicated French), 15 reported to have basic knowledge of a foreign language (four of those indicated French) and three reported to speak a foreign language fluently (all indicated Spanish). Among the French participants, three people reported to have studied a foreign language at school, 23 reported to have basic knowledge of a foreign language, and 78 reported to speak a foreign language fluently. In total, 76 French participants indicated to have more or less advanced knowledge of English, though there were no French-English bilinguals in the sample. We checked if having foreign language experience with French (for English participants) or English (for French participants) had an influence on the perceptual responses but found no evidence in support of an effect.

Informed consent was obtained from all participants prior to the start of the experiment. The study ran at the Linguistics Labs of the University of Konstanz and was approved by the Institutional Ethics Review Board (approval date: 04/02/2021). All methods were performed in accordance with the relevant guidelines and regulations.

### Materials

**Fig. 1:**
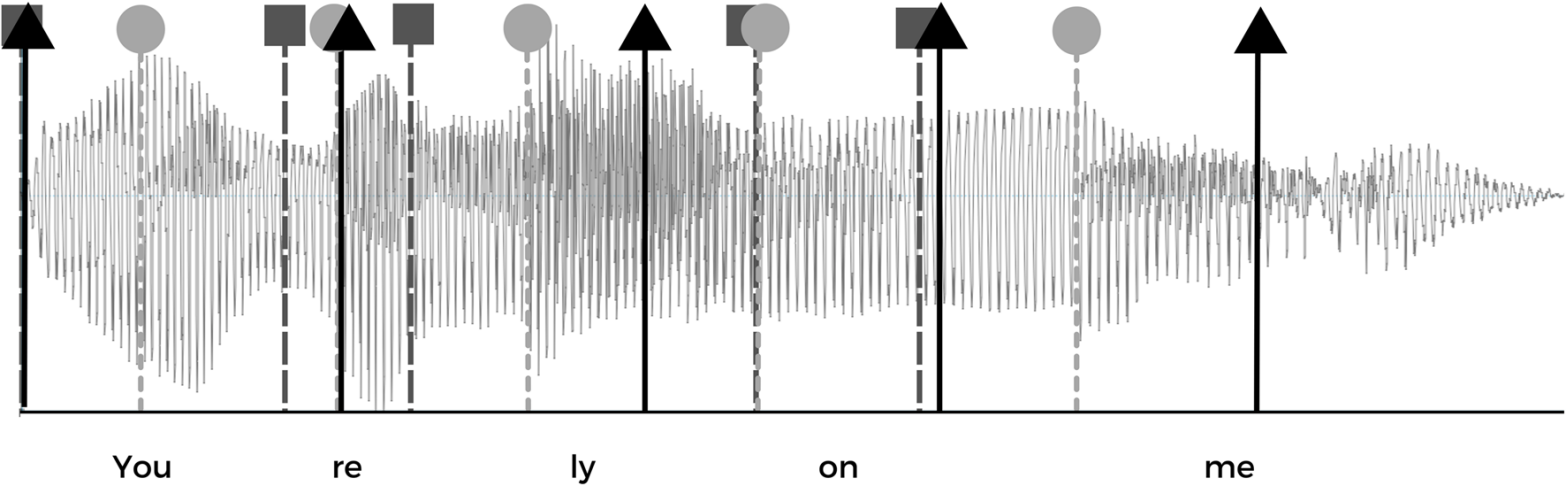
Example of an English sentence (“You rely on me”) with three locations of superimposed drumbeats used in the experiment: (1) syllable onsets (indicated by *dark grey squares*), (2) vowel onsets (indicated by *light grey circles*), and (3) isochronous intervals (indicated by *black triangles*)

For each language (English and French), ten naturally spoken sentences varying in length from 4 to 11 syllables were chosen from an existing database (Rathcke et al., 2021). The sentences were annotated in Praat (Boersma, 2001) by the second author who manually identified vowel and syllable onsets. A Praat script was then used to extract the time points of the annotated onsets. Drum beats of short (55 ms) duration represented by the sound of a drum (recorded from a synthesizer) were then added to the sentences at the time points corresponding to either syllable or vowel onsets. The drumbeats overlaid each sentence; their total number amounted to the number of syllables per sentence. In addition, one regularized drumbeat version of each sentence was created as a control condition, with drumbeats overlaying each sentence at isochronous intervals and matching the duration of the sentence as well as the total number of its syllables. This procedure resulted in three versions of each sentence for all languages, resulting in a total of 63 stimuli. The loudness of the drumbeats and the sentences were set to a comparable level, whereby the acoustic and perceptual tuning was combined. First, a Praat script scaled both sounds to the same absolute peak; second, the two sounds were perceptually compared and the louder-perceived sound was step-by-step re-scaled until both sounded equally loud to the experimenters. The three versions of each sentence contained the same number of drumbeats but differed with regards to the drumbeat location, occurring at (1) syllable onsets, (2) vowel onsets, or (3) isochronous intervals (see Fig. 1). They were paired with the non-manipulated recording to create an experimental trial. Examples of each stimuli version in both languages can be found on https://osf.io/m2p3x/view_only=bc5d958246104df2b17e838b659272fb (link to be made public after peer-review).

### Procedure

The experiment was conducted online, with an individual session lasting 15–20 min. The cross-linguistic perception task was run on Gorilla (www.gorilla.sc) (Anwyl-Irvine et al., 2020, 2021) and the musical task was run on a local server (Harrison & Müllensiefen, 2018a). Participants were recruited and remunerated via the Prolific Academic platform (www.prolific.co). They were instructed to use a tablet or a laptop computer while taking part and to play the sounds of the experiment through the built-in speakers of their devices (no wireless earphones or headphones were allowed). Participants were recruited and tested in their native language (English or French). The session started with the background questionnaire, then moved to the cross-linguistic perception task and ended with the beat perception test. Once participants reached the end of the cross-linguistic perception task on Gorilla, a new link open opened on a separate page running the beat perception test of CA-BAT (Harrison & Müllensiefen, 2018a).

At the beginning of the cross-linguistic perception task, participants were instructed that they would listen to sentences spoken in different languages (including their own native language). On each trial, they first heard a non-manipulated version of a sentence, then the same sentence overlaid with drumbeats in one timing condition (inter-syllabic, inter-vocalic, or isochronous drumbeats). Participants’ task was to indicate as quickly as possible if the drum matched the beat of the sentence or not, by rating the drum beats as being the same or different from the sentence beat. Participants listened to three blocks of 21 trials (or 63 trials in total) preceded by two practice trials. The practice trials were not repeated in the main task.

The Computerised Adaptive Beat Alignment Test (CA-BAT) (Harrison & Müllensiefen, 2018b, a) was used to test individual beat perception ability. The results of the test have been shown to correlate with a range of timekeeping abilities (Harrison & Müllensiefen, 2018b, a) and were expected to rely on similar temporal processing mechanisms as would be needed for the cross-linguistic task described above. CA-BAT requires participants to listen to a musical extract with a superimposed metronome beat and to indicate whether or not the metronome matches the beat of the music. The test is adaptive, meaning that the location of the metronome beats changes depending on participants’ performance. The results of the test are given as a *z*-scored BAT value, normed with reference to the sample of the original study that consisted of 197 participants (87 female) aged between 18 and 75 (mean age, 26 years) (Harrison & Müllensiefen, 2018b). Accordingly, a score around 0 reflects an average beat perception ability, scores above 0 indicate an above-average ability, and scores below 0 a below-average ability. The BAT scores of the present sample showed a normal distribution with a slight skew toward above-average BAT scores with the average BAT ability in the English sample being slightly higher than in the French sample (ENG-FRE: $\beta$ = 0.08, SE = 0.04, CI [0.02, 0.14], ER = 58.88, PP = 0.98).

### Statistical analysis

The data were analyzed using Bayesian multilevel regression models that were performed in the statistical program R (R Core Team, 2021). We used the brms package and Stan for the calculations (Bürkner, 2017, 2018; R Core Team, 2021). We tested the effects of two predictor variables (drumbeat timings, BAT scores) on the likelihood of the perception of sentence rhythm as identical to, or different from, the given drumbeat timing. The dependent variable was coded as a binary response (same = 0, different = 1) and modeled using a Bernoulli distribution. The analysis is divided into two sets of models: (1) native language models and (2) foreign language models. The first set of models analyzed the interaction between *Drumbeat timing* and *BAT ability* on participants’ responses to their native language trials. The second set of models analyzed the interaction between *Drumbeat timing* and *BAT ability* on participants’ responses to their foreign language trials (i.e., English listeners responding to French sentences and vice versa).

For each of these models, we used a weakly informative prior with a Student’s *t*-distribution and 3 degrees of freedom, a mean of 0 and a scale of 1. For our hypothesis testing, we quantify the strength of evidence using evidence ratios (ER). ERs represent the posterior probability that the effect is in the hypothesized direction divided by the posterior probability that the effect is in the opposite direction (Smit et al., 2022). We consider ERs > 19 to indicate ‘strong evidence’, given the directional hypothesis test (Makowski et al., 2019).

**Fig. 2:**
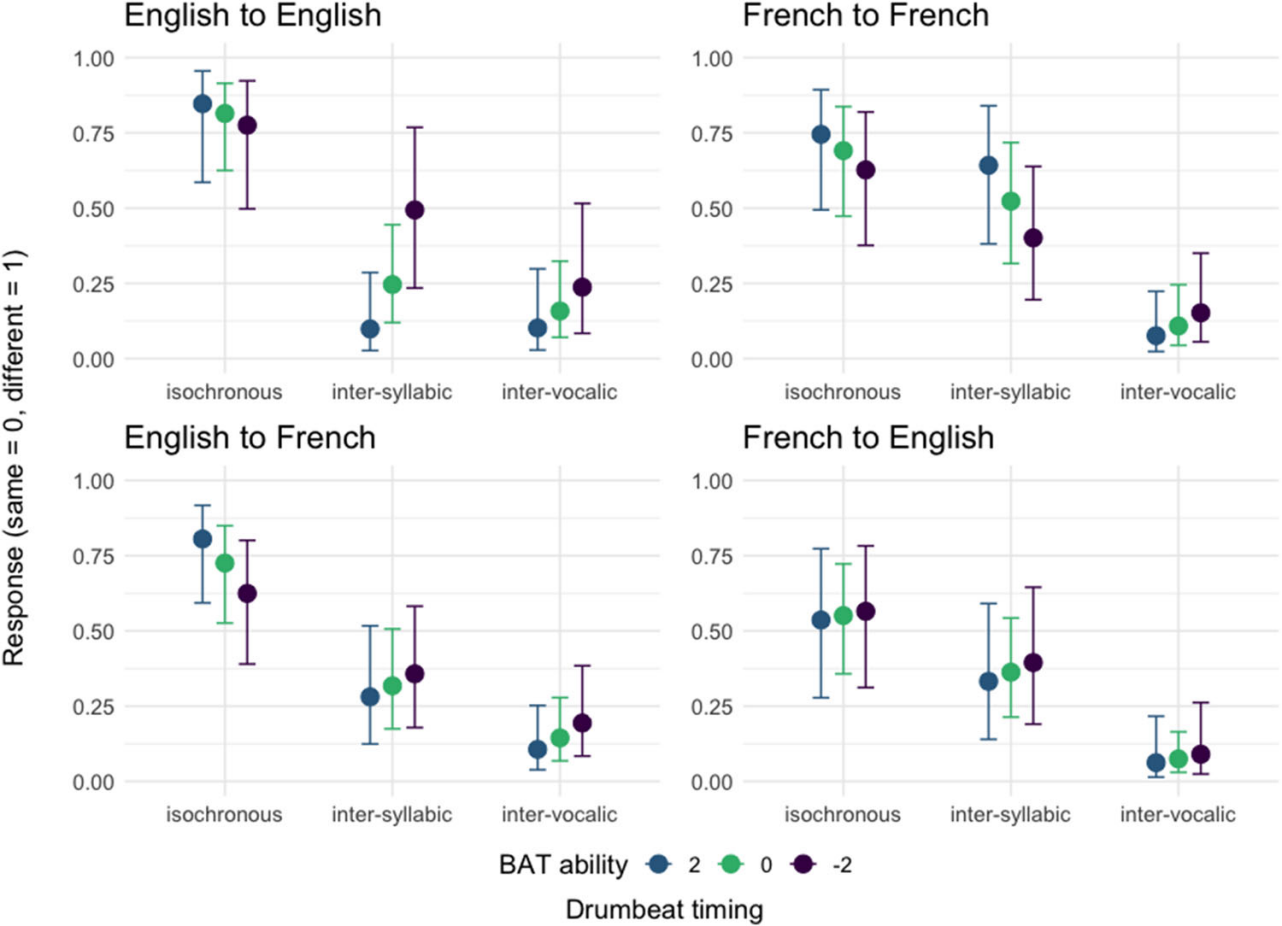
Conditional effects for the interaction between drumbeat timing and BAT ability for English and French participants listening to their native language trials (*top panels*) versus English and French participants listening to their foreign language trials (*bottom panels*)

Figure 2 displays the results of these analyses, with top panels showing the conditional effects of the interaction between *Drumbeat timing* and *BAT ability* for both English and French participants listening to their native language trials and bottom panels showing the effects for the foreign language trials. The data and scripts associated with the analyses can be found on https://osf.io/m2p3x/view_only=bc5d958246104df2b17e838b659272fb (link to be made public after peer review).

## Results

### Native language models

Focusing first on the performance of English participants on native language trials, we found strong evidence that the participants with an average BAT score were more likely to rate *inter-syllabic* and *inter-vocalic* drumbeats as perceptually equivalent to the sentence beat, compared to the *isochronous* drumbeat timing. There was insufficient evidence to document a difference between the *inter-syllabic* and the *inter-vocalic* timings. We further found strong evidence for a positive effect of BAT ability on perceiving the *inter-syllabic* timing similar to the sentence beat but no evidence for an effect of BAT ability on either the *inter-vocalic* or the *isochronous* timing.

Turning next to the performance of French participants on native language trials, we found strong evidence that the participants with an average BAT score were more likely to rate the *inter-vocalic* drumbeats as being more similar to the sentence beat than the drumbeats with the *isochronous* or *inter-syllabic* timing. There was insufficient evidence to document a difference between *inter-syllabic* and *isochronous* drumbeats. Again, an effect of BAT ability could only be observed for the *inter-syllabic* timing, though it showed in the opposite direction to the effect observed in the English data. That is, we found strong evidence for a negative effect of BAT ability on perceiving the *inter-syllabic* timing similar to the sentence beat.

In summary, English listeners tended to equally map sentence beat to syllable and vowel onsets whereas French listeners tended to only map the beat onto vowel onsets (see Table 1 for the full results), with these tendencies being enhanced in listeners with a high beat perception ability.

**Table 1:** Estimate = mean of the effect’s posterior distribution. 90% CI = 90% credibility intervals. ER = evidence ratio, or the odds that the effect is in the direction specified by the hypothesis. PP = the posterior probability

	Hypothesis	Estimate	Est. Error	[90% CI]	ER	PP
English to English						
*Mean BAT ability*	inter-syllabic - isochronous < 0	-2.57	0.65	[-3.62, -1.49]	3999.00	1.00
	inter-vocalic - isochronous < 0	-3.13	0.67	[-4.20, -2.01]	6665.67	1.00
	inter-syllabic - inter-vocalic > 0	0.56	0.62	[-0.44, 1.57]	4.85	0.83
*Effect of BAT ability*	isochronous > 0	0.12	0.23	[-0.26, 0.50]	2.24	0.69
	inter-syllabic < 0	-0.55	0.23	[-0.94, -0.18]	162.93	0.99
	inter-vocalic < 0	-0.26	0.23	[-0.64, 0.13]	6.35	0.86
French to French						
*Mean BAT ability*	inter-syllabic - isochronous < 0	-0.69	0.54	[-1.59, 0.21]	9.36	0.90
	inter-vocalic - isochronous < 0	-2.91	0.66	[-3.97, -1.80]	19999.00	1.00
	inter-syllabic - inter-vocalic > 0	2.22	0.63	[1.19, 3.25]	908.09	1.00
*Effect of BAT ability*	isochronous > 0	0.13	0.15	[-0.11, 0.38]	4.56	0.82
	inter-syllabic > 0	0.25	0.14	[0.01, 0.48]	22.64	0.96
	inter-vocalic < 0	-0.20	0.17	[-0.47, 0.08]	7.47	0.88

**Table 2:** Estimate = mean of the effect’s posterior distribution. 90% CI = 90% credibility intervals. ER = evidence ratio, or the odds that the effect is in the direction specified by the hypothesis. PP = the posterior probability

	Hypothesis	Estimate	Est. Error	[90% CI]	ER	PP
English to French						
*Mean BAT ability*	inter-syllabic - isochronous < 0	-1.73	0.56	[-2.62, -0.79]	383.62	1.00
	inter-vocalic - isochronous < 0	-2.72	0.60	[-3.68, -1.72]	19999.00	1.00
	inter-syllabic - inter-vocalic > 0	1.00	0.57	[0.07, 1.92]	24.84	0.96
*Effect of BAT ability*	isochronous > 0	0.23	0.14	[0.01, 0.45]	21.57	0.96
	inter-syllabic < 0	-0.09	0.15	[-0.32, 0.15]	2.63	0.72
	inter-vocalic < 0	-0.18	0.15	[-0.43, 0.08]	6.87	0.87
French to English						
*Mean BAT ability*	inter-syllabic - isochronous < 0	-0.77	0.50	[-1.58, 0.05]	15.38	0.94
	inter-vocalic - isochronous < 0	-2.73	0.61	[-3.72, -1.72]	19999.00	1.00
	inter-syllabic - inter-vocalic > 0	1.96	0.59	[1.00, 2.94]	908.09	1.00
*Effect of BAT ability*	isochronous < 0	-0.03	0.19	[-0.34, 0.28]	1.24	0.55
	inter-syllabic < 0	-0.07	0.19	[-0.38, 0.24]	1.80	0.64
	inter-vocalic < 0	-0.11	0.27	[-0.55, 0.33]	1.91	0.66

### Foreign language models

Finally, we rerun the Bayesian models with the data collected from the trials in which English and French participants were listening to sentences in their foreign language (see bottom panels of Fig. 2). The results of these models were, to some extent, quite similar to the results of the native language models reported above.

English participants with an average BAT ability were more likely to rate *inter-syllabic* and *inter-vocalic* (rather than *isochronous*) drumbeats as perceptually equivalent to the sentence beat in French stimuli. Again, we found insufficient evidence for a difference between the perception of the *inter-syllabic* and the *inter-vocalic* drumbeat timing. As far as BAT ability is concerned, we now only found strong evidence for a negative effect on the perception of *isochronous* drumbeats, with high BAT ability listeners rating the beat of French sentences as less similar to the *isochronous* timing.

For French participants at an average BAT ability, again, we found strong evidence that they were more likely to rate *inter-vocalic* (compared to *isochronous* condition and *inter-syllabic*) drumbeats as perceptually equivalent to the beat of English sentences. There was also moderate evidence to document a difference between their perceptual ratings of *inter-syllabic* and *isochronous* drumbeats (see Table 2 for the full results). As far as BAT ability is concerned, we found no evidence for its role in the French participants’ performance on foreign trials.

In summary, English listeners mapped sentence beat in a foreign language onto syllable and vowel onsets, displaying perceptual equivalence patterns that were comparable across their native and foreign language trials. Similarly, French listeners mapped the beat in their foreign language primarily onto vowel onsets as they did on their native language trials. The beat perception ability only played a role for English but not French listeners. English natives with a high beat perception ability showed a diminished tendency to regularize the beat in their foreign language, as compared to English natives with a low beat perception ability who perceptually regularized more (Rathcke et al., 2021; Benadon, 2014).

## Discussion

This present study investigated the perception of speech rhythm in native listeners from two prosodically distinct languages – English and French. The participants rated perceptual equivalence of sentences spoken in their native and foreign language, comparing natural and superimposed drumbeat versions of each sentence. The drumbeat versions of sentences contained one drumbeat per syllable and varied in the timing of beat locations, augmenting either syllable or vowel onsets or being isochronously distributed across sentence duration. Perceptual performance of the two listener groups differed quite remarkably: while English listeners showed no strong preference for either syllabic or vocalic beat onsets, French listeners preferred drumbeats to coincide with vowel onsets only. These perceptual preferences were enhanced with increasing beat perception ability and transferred to the listeners’ foreign language. The finding provides strong support for the linguistic-bias hypothesis as general beat perception ability matters less than the native language background in the explanation of the current results. It further speaks to recent evidence that highlights a crucial contribution of cultural and linguistic experience to rhythm perception in both language and music (Cameron et al., 2015; Zhang et al., 2020).

At the same time, there are also some discrepancies between the study and the specific assumptions we made with reference to previous research (Varghese et al., 2022; Rathcke et al., 2021) and theoretical tradition of rhythm classes (Abercrombie, 1967; Pike, 1945). In particular, English listeners were expected to map the sentence beat onto vowel onsets, given that vowel onsets act as the attractors of rhythmic movement during sensorimotor synchronization (Rathcke & Lin, 2023; Rathcke et al., 2021), and French listeners were primarily expected to map the beat onto syllable onsets, given that their “syllable-timed” rhythm class and enhanced cortical tracking at the syllable rate (Varghese et al., 2022; Abercrombie, 1967; Pike, 1945). Neither of these detailed predictions was borne out by the present data which, on the one hand, reinforces previous criticisms of the rhythm-class idea (Arvaniti & Rodriquez, 2013; Arvaniti, 2009; Aubanel & Schwartz, 2020; Rathcke & Smith, 2015) and, on the other hand, indicates the need for a methodologically integrated approach to the study of speech and language rhythm.

The nature of a task – i.e., perception–action coupling during sensorimotor synchronization with natural speech (Rathcke & Lin, 2023; Rathcke et al., 2021), passive listening to a series of synthesized, concatenated syllables (Varghese et al., 2022), or perceptual equivalence ratings comparing natural and rhythmically enhanced speech as in the present study – seems to bring about differences in the temporal resolution of the rhythmic structure. The experimental design of the present study was inspired by similar investigations of rhythm processing in music (Harrison & Müllensiefen, 2018b, a), targeting only one – the perceptual - component of the multidimensional complex that is rhythmic skill (Fiveash et al., 2022). However, a due consideration of the multidimensionality of rhythm (which includes both perception, production, and other abilities) is key to resolving the controversy surrounding rhythm in speech and language (Roach, 1982).

The results of the present study have implications for the interpretation of previous findings comparing cortical tracking performance of French and English listeners (Varghese et al., 2022). If French listeners have a strong preference for sampling syllables at one specific time point (namely the vowel onset), this perceptual trend may be reflected in an enhanced cortical tracking at syllable rate as compared to English listeners who have a less pronounced perceptual preference for rhythmic sampling at one specific time point of a syllable. Only a combination of methodologies can help with testing this hypothesis.

Finally, we find little evidence in support of the innate cognitive skill hypothesis (Merchant et al., 2015; Honing, 2013; Nettl, 2000). Beat perception ability played a major role in the participants’ ratings of auditory stimuli in their native language but not so much in their foreign language. This ability was measured using CA-BAT (Harrison & Müllensiefen, 2018b, a), with the study participants showing substantial variability in their BAT scores similar to what has been documented in previous research (Bégel et al., 2017; Phillips-Silver et al., 2011; Bekius et al., 2016), which may have been a consequence of the task being administered online instead of in the laboratory (Harrison & Müllensiefen, 2018b). Here, we observed a trend toward perceptual regularization of the rhythmic structure of natural sentences in listeners with a low beat perception ability, though this trend was limited to English listeners rating foreign speech stimuli. While it has long been known that the perception of time intervals in speech deviates from their actual acoustic durations toward more regular timings (Darwin & Donovan, 1980; Scott et al., 1985), a comprehensive explanation of the perceptual regularization effect is still lacking. The present finding suggests that both individual listener traits (i.e., beat perception ability) and auditory stimulus complexity (i.e., native vs. foreign language) might contribute to the origin of the perceptual effect. Regularization prevails in low-ability listeners perceiving auditory prompts that are more difficult for them to parse, such as sentences spoken in a foreign language.

## Conclusion

In conclusion, we found that primarily native language background and, to a lesser extent, individual beat perception ability impact rhythm perception in native and foreign speech. These results make a major contribution to the ongoing discussion of how rhythm processing in language may be shaped by top-down processes arising from experiential priors and bottom-up acoustic features identifiable in auditory signals.

## Declarations

### Open Practices Statement

The data and materials that support the findings of this study are available on https://osf.io/m2p3x/view_only=bc5d958246104df2b17e838b659272fb
